# Clinical Assessment of Central Blood Pressure

**DOI:** 10.2174/157340212800840708

**Published:** 2012-05

**Authors:** Hiroshi Miyashita

**Affiliations:** Jichi Medical University Health Care Center, Jichi Medical University School of Medicine, Tochigi, Japan

**Keywords:** Arterial applanation tonometry, brachial cuff blood pressure, calibration method, central aortic blood pressure, estimation method, generalized pressure transfer function, radial second systolic pressure.

## Abstract

Central aortic blood pressure (CBP) is increasingly considered a better cardiovascular prognostic marker than conventional cuff brachial blood pressure. Because CBP cannot be directly measured noninvasively, it has to be estimated from peripheral pressure pulses. To assess estimated CBP appropriately, the accuracy and features of the estimation method should be considered. The aim of this review is to provide basic knowledge and information useful for interpreting and assessing estimated CBP from a methodological point of view. Precise peripheral pressure pulse recording has been enabled by the introduction of arterial applanation tonometry, for which the radial artery may be the optimal site. An automated tonometry device utilizing a sensor array is preferable in terms of reproducibility and objectivity. Calibration of a peripheral pressure waveform has unresolved problems for any estimation method, due to imperfect brachial sphygmomanometry. However, if central and peripheral pressure calibrations are equivalent, two major methods to estimate CBP—those based on generalized pressure transfer function or radial late systolic pressure—may be comparable in their accuracy of CBP parameter estimation.

## INTRODUCTION

It has been considered basic knowledge, usually described in textbooks of circulatory physiology, that peripheral systolic blood pressure (SBP) is higher than central aortic SBP due to pulse pressure (PP) amplification [1, 2]. In the clinical treatment of hypertension, however, this phenomenon has long been ignored, and brachial cuff sphygmomanometric blood pressure (BP) has been used as a reliable alternative to central BP (CBP) until recently. The results of the Conduit Artery Function Evaluation (CAFE) study [3] reminded clinicians of the importance of CBP. Those results demonstrated significant differences in CBP (central SBP and PP) between patient groups treated with different antihypertensive regimens even though peripheral BP levels were comparably lowered, and suggested the potential superiority of CBP to cuff brachial BP in cardiovascular prognostic predictive value in hypertensive patients. 

Advances in knowledge and technology were crucial to enabling this study; these include pulse wave analysis (PWA) utilizing pressure transfer function-based CBP waveform estimation in concert with precise applanation tonometry of the radial artery. Thereafter, other methods and devices became also commercially available to assess CBP. Because CBP cannot, in principle, be directly measured noninvasively, it should be estimated from peripheral pressure waveforms. Hence, to assess CBP appropriately, the accuracy and features of each estimation method should be considered. 

This review aimed to provide basic knowledge and information useful for interpreting and assessing estimated CBP, focusing on the methodology and devices for CBP estimation used in clinical studies as well as in clinical practice.

## OVERVIEW OF CBP ESTIMATION METHODS

In most recent clinical studies, SphygmoCor^®^ （AtCor Medical, Australia） or HEM-9000AI^®^ (Omron Healthcare, Japan) was used to estimate CBP. These devices became available after the beginning of this century. In earlier studies, the calibrated carotid pulse waveform was used as an alternative to an aortic pressure wave [4, 5]. In a recent report on the Framingham study [6], this carotid pulse wave method was still used, probably because the longitudinal study began before the other methods were available. Currently, five categories of CBP estimation methods have been available, as shown in Table **1**. In four of these methods, a peripheral artery pulse waveform is recorded by applanation tonometry, which was first applied to arterial pulse wave recordings by Kelly *et al*. [4, 7]. 

More recently, on the other hand, several oscillometric devices, designed to assess CBP and arterial stiffness-related indexes in addition to ambulatory BP monitoring (ABPM), have appeared. In some of these devises such as Mobil-O-Graph^®^ (APC Cardiovascular, United Kingdom) and BPLab^®^ + Vasotens^®^ software (OOO Petr Telegin, Russia), ordinary oscillometric pulse volume recording (diastolic oscillometry) data are fed into a certain transfer function to estimate a central aortic pressure waveform. In Arteriograph^®^ (MedExpert, Hungary) and BPPlus CardioScope^®^ + VasomonR^®^ software (PulseCor, New Zealand), oscillometric technique has been modified to acquire suprasystolic recordings of oscillometric brachial pulse waves to estimate CBP parameters. “Suprasystolic” or “stop-flow” recordings are made with a cuff pressure above SBP so that the brachial artery is totally occluded [8]. These devices may offer the advantage of acquiring CBP as well as ABPM easily at one time if the oscillometric pulse waveform recording is fully validated. Although some validation studies have already been published on Arteriograph^®^ [9, 10], BPPlus^®^ [8], and BPLab^®^ [11], and although the physical arterial model that the method is based on is correct, the theoretical validity of the use of a simple cuff as a pressure sensor is not fully understood. Moreover, demonstrative clinical data supporting its accuracy seem to be inadequate. In these reported validations, the limits of agreement with a standard method seem larger than expected. Although these devices are also expected to offer the advantage of ambulatory assessment of CBP, their clinical validity has not been fully evaluated. Hence, these newer devices are still thought to be evaluated as valid clinical tools [12].

The following discussion will focus on well-validated tonometry-based CBP estimation methods.

## THE PRINCIPLES OF APPLANATION TONOMETRY AND TONOMETRIC SENSOR OPERATION

**Fig. (1):**
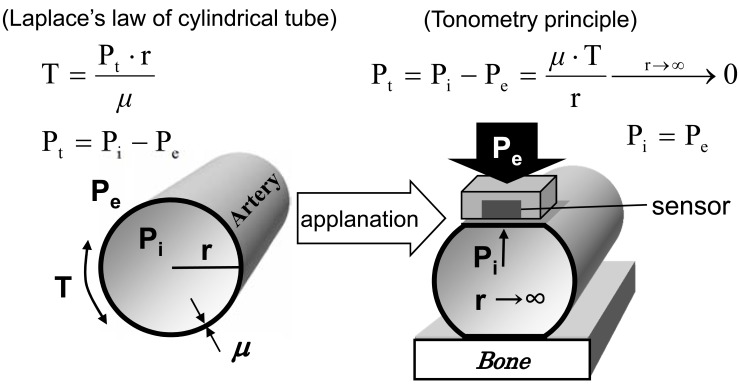
**Principle of arterial applanation tonometry.** P_e_ = external pressure; P_i_ = internal pressure; P_t_ = transmural pressure; r =radius of wall curvature; T = wall tension; *µ*= wall thickness.

The law of Laplace shows a relationship between wall tension (T) and transmural pressure (P_T_) and the radius of a cylindrical thin wall tube. Based on this law, when a pressure sensor applanates the tube wall (the radius of the wall curvature becomes infinity) by external pressure (P_e_), P_e_ is identical to internal pressure (P_i_) so that the sensor output exactly reflects P_i_ (Fig. **1**). For valid tonometry, an applanated tube wall should stably cover the sensor’s whole surface. Requirements for effective applanation of the arterial wall by a tonometry sensor include:

- The artery at the measurement site runs shallow beneath the skin and is fixed on a hard tissue such as a bone.
- The sensor is always smaller than the applanated area of the arterial wall along the cardiac cycle.
- The sensor’s position is exactly fixed and is not influenced by physical movement during measurement.

The radial artery can satisfy all these conditions. However, it is difficult to apply valid applanation tonometry to carotid and brachial arteries, as they are usually buried in soft tissues under the skin and not fixed on a bone. The carotid artery has an additional difficulty, which is movement in the surrounding tissues due to respiration [13]. Further, there might be risks that the hold-down pressure of the sensor probe causes atherosclerotic plaque rupture, and that breath holding as well as mechanical stimulation of the baroreceptor might induce BP fluctuation during a measurement. 

As shown in Table **1**, two types of tonometry sensor structure are used: single and arrayed. In the case of a single sensor, the operator holds a sensor probe manually, and should select a measurement site and adjust the hold-down pressure of the sensor to obtain an optimal pulse wave recording by inspecting the monitored waveform. Hence the excessive bias relating to the operator’s skill and subjective data selection may be inevitable. In this case, a reproducibility study is essential for each operator in order to guarantee the measurement quality.

In contrast, an arrayed sensor, once set on the subject’s wrist, is servo-controlled to optimize the hold-down pressure in order to attain effective applanation of the artery and automatically select a sensor element outputting the highest-quality tonometric waveform. There is no room for subjective data selection or dependence on the operator’s skill. This type of sensor is adopted only in the HEM-9000AI^®^ device. In addition, the full measurement process is semi-automated, including calibration by brachial BP with an inbuilt oscillometric sphygmomanometer. Although the widely used SphygmoCor^®^ device uses a single sensor for measurement, it is notable that some validation studies for generalized pressure transfer functions (GTF) have utilized an automated tonometry system based on an arrayed sensor along with subsequent offline GTF-based CBP estimation in order to avoid issues relating to a manually operated single sensor [14, 15]. 

In a recently developed device, BPro^®^ (HealthSTATS, Singapore), a modified tonometry sensor is embedded in a wrist strap, which is simply fixed to the radial artery with the wrist strap. Although little information about the details of the “modified” tonometric sensor have appeared, at least from published reports or the manufacturer’s web site, a recent validation study showed acceptable results [16]. This wristwatch-like device is small enough to wear around the wrist. It is expected to enable ambulatory arterial tonometry, which would constitute an important advantage, but it also has the potential drawback of an inconstant positional relationship between the heart and the measurement site, which would introduce the influence of hydrostatic pressure alterations on the BP level, potentially leading to excessive errors or inaccuracy of ambulatory CBP assessment.

## CALIBRATION OF PERIPHERAL PULSE WAVEFORM

**Fig. (2):**
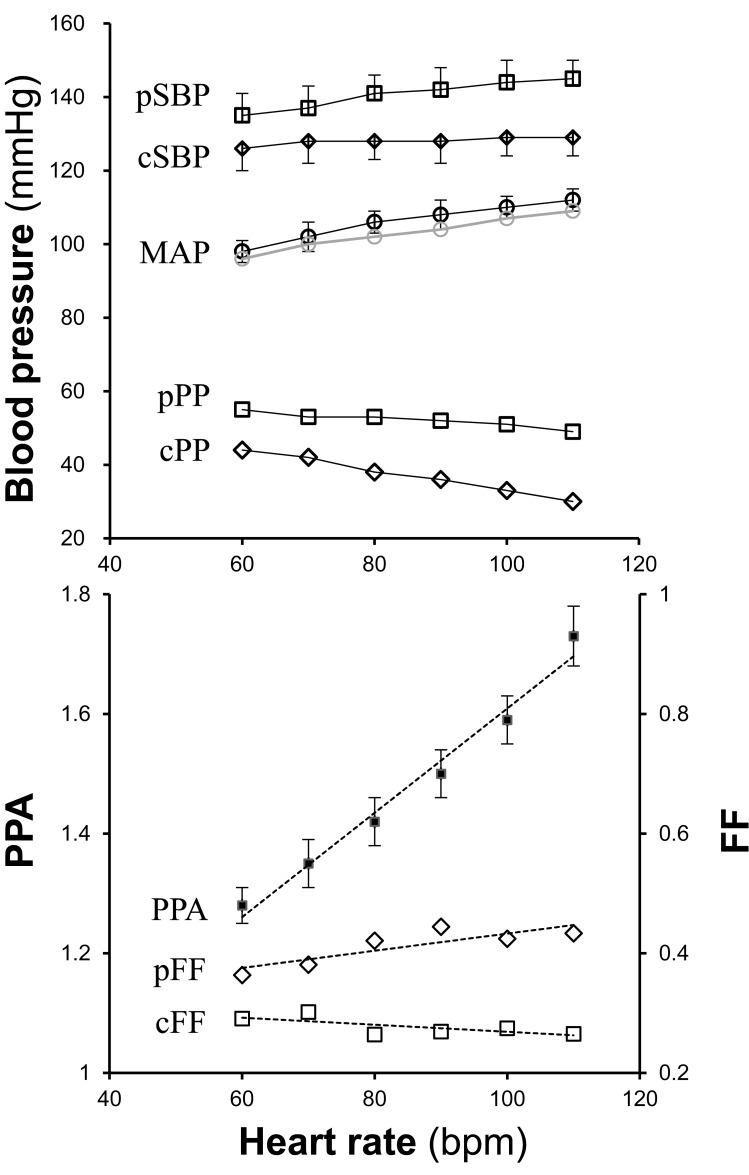
**Heart rate dependence of central blood pressure and pulse pressure amplification (PPA).** Published pacing study data [22] in regard to PPA and FF are re-plotted. The study was conducted to investigate the influence of heart rate on the central augmentation index (cAI) derived from GTF-based estimation. The original paper might have reported peripheral and estimated central MAPs in reverse in the original Table **2**, in which peripheral MAP is abnormally higher than central MAP (shown as a gray series in the upper line graph). Using the reported MAPs directly to calculate FFs led to inconsistency with the physiological relationship between cFF and pFF. Therefore, the lower diagram is plotted based on reversed MAPs; i.e., peripheral MAP is used as central MAP and *vice versa.* cFF = central form factor; cPP = central pulse pressure; cSBP = central systolic blood pressure; FF = form factor; MAP = mean arterial pressure; pFF = peripheral form factor; PPA = pulse pressure amplification; pPP = peripheral pulse pressure; pSBP = peripheral systolic blood pressure.

Cuff brachial BP has been used in common for the calibration of a peripheral tonometric pulse waveform, which is the basis of all CBP estimation methods (Table **1**). A calibration algorithm is different between pulse waveform recording sites depending on the difference in PP (i.e., PP amplification; PPA) from the brachial site. When PP does not significantly differ from brachial PP as the radial artery, the peak and the bottom of a waveform are simply adjusted to brachial SBP and diastolic BP (DBP), respectively. In the case of the carotid artery, where PPA is usually significant, the mean and the bottom of the pulse waveform are adjusted to brachial mean arterial pressure (MAP) and DBP, respectively, based on the observation that no significant pressure drop occurs within the conduit arteries [2]. In this case, the peak BP is estimated according to the form factor (FF) = (MAP-DBP)/PP. As an exact MAP cannot be obtained by sphygmomanometry, it is usually calculated with conventionally assumed FF (=1/3). This assumption can affect the accuracy of this calibration method [13]. Taking this into account, FF of the brachial artery was actually measured by applanation tonometry in some studies [17, 18]. These studies has been criticized [19, 20] on the one hand and supported [21] on the other, as the reported actual FF of the brachial artery was largely different from invasively measured data reported previously, which is the basis of the assumption that there is no significant PPA between brachial and radial arteries. The chief reason for the criticism was the poor quality of the tonometric waveform at the brachial site, where conditions for arterial applanation tonometry might be suboptimal. Although this issue may still be controversial, the attempt to confirm actual FF has been favorable. FF as well as PPA may not be constant even in an individual because these properties depend at least on heart rate [18]. Data reported in a pacing study by Wilkinson *et al* [22, 23] clearly demonstrated the heart rate dependence of PPA and FF (Fig. **2**).

For a century after its introduction, brachial sphygmomanometry has been the standard clinical measure and basis of evaluation and treatment of hypertension. However, its inaccuracy became recognized when CBP estimation accuracy was assessed. This required a direct comparison between invasively measured (actual) CBP and estimated CBP derived from tonometric pulse waveforms calibrated to cuff brachial BP [24-26]. However, there has been no alternative noninvasive means to acquire absolute BP levels for calibration.

## CBP ESTIMATION ALGORITHMS

### Substitution of Calibrated Carotid Pulse Waveform to Central Aortic Pressure Wave

The carotid artery is anatomically adjacent to the central aorta with no significant PPA between the two sites; i.e., the peak systolic pressures at both sites are nearly identical. Therefore, a carotid artery pulse waveform calibrated by the method described above is used as an alternative to a central aortic pressure waveform to measure CBP parameters such as cSBP [4, 27]. However, as discussed above, the validity of the MAP/DBP (or FF)-based calibration method has not been fully established. Besides, there are excessive disadvantages related to the applanation tonometry of the carotid artery in addition to the inaccuracy related to cuff brachial blood pressure measurements for calibration, which is common to all methods. Therefore, the calibration of tonometric carotid pulse waveform may be regarded as a legacy method used for research purposes before other methods using radial artery tonometry became available. 

Generally speaking, most European researchers attach much importance to carotid-femoral pulse wave velocity. Some of them seem to prefer and rely on this carotid pulse wave-based method to estimate CBP, so they regard the derived CBP estimates as the standards against which to test other estimation devices, such as the GTF-based SphygmoCor^®^ [17, 18], possibly because of the practical and/or ethical advantages it offers compared with invasive methods. 

### GTF-based Method

The generalization of pressure pulse transduction properties, expressed as a transfer function, between the central aorta and peripheral upper limb arteries was first proposed by Karamanoglu *et al*. [28] based on their observation that individual differences in transduction properties were small for the lower frequency range up to 3 Hz, which includes 90% of frequency components. The transfer function is a system function that identifies a linear, time-invariant system (the upper limb arterial system in this case), which has been a common fundamental theory for engineering purposes [29], as the relationship between input (aortic pressure wave) and output (peripheral pressure wave) signals of the system in the frequency domain. Recent progress in sensor as well as computer technologies has enabled the clinical application of this mathematical method. 

**Fig. (3):**
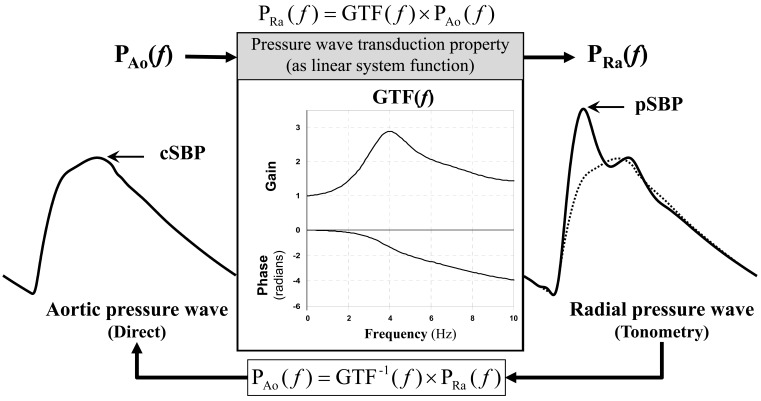
**Central aortic blood pressure waveform estimation from peripheral radial pressure wave based on a generalized pressure transfer function (GTF).** P_Ao_ = aortic pressure wave; P_Ra_ = radial artery pressure wave; cSBP = central systolic blood pressure; pSBP = peripheral systolic blood pressure. “(*f*)” indicates a function of frequencies. For waveform comparison, the estimated central aortic pressure waveform (broken line) is superimposed on the radial pressure waveform.

An averaged aorto-radial pressure transfer function (PTF) determined using data obtained from a certain population is used as a generalized PTF (GTF) to calculate a central aortic pressure waveform, which in turn is used to determine CBP estimates (Fig. **3**). During the procedure to determine a practical GTF, further investigation revealed that the autoregressive exogenous (ARX) model-based parametric PTF in the reverse causal direction (i.e., radial to aorta) is better than the conventional Fourier transform-based non-parametric aorto-radial PTF [30].

The SphygmoCor^®^, the first device that employs this method, was used in a large clinical trial such as the CAFE study [3] after extensive validation studies [14, 15, 31, 32]. This device seems to be regarded as the *de facto* standard of CBP estimation. However, it should be noted that the results of validation studies showed acceptable estimation accuracy only for CBP parameters such as cSBP and cPP. Although a feature of this method is the capability to obtain a full waveform of central aortic pressure, the estimated aortic waveform was not precise enough for detailed wave contour analysis requiring higher-frequency components, such as augmentation index measurement [14, 33]. 

### NPMA Method

Recently, the n-point moving average (NPMA) method has been applied for estimation of cSBP and extensively validated [16]. This algorithm is included in the A-PULSE CASP^®^ (HealthSTATS) software provided in combination with the BPro^®^ device. The NPMA algorithm is a kind of digital low-pass filter usually used for smoothing waveforms to eliminate high-frequency noise. In the GTF-based method, the inverse of GTF used to estimate CBP from peripheral pressure pulse has low-pass characteristics up to about 4~5 Hz (corresponding to the peak gain frequency shown in Fig. **3**). It is therefore taken for granted that the estimation method works well if the low-path characteristics are optimized. Ideally, the optimization is adjusted to the inverse of the individual pressure transfer function. However, as individualized optimization is practically impossible, as is the case with GTF, an optimal denominator for the moving average was determined empirically using validation data from a selected population [16]. Therefore, the accuracy of this method cannot be superior to that of the GTF-based method. 

### SBP2-based Method

**Fig. (4):**
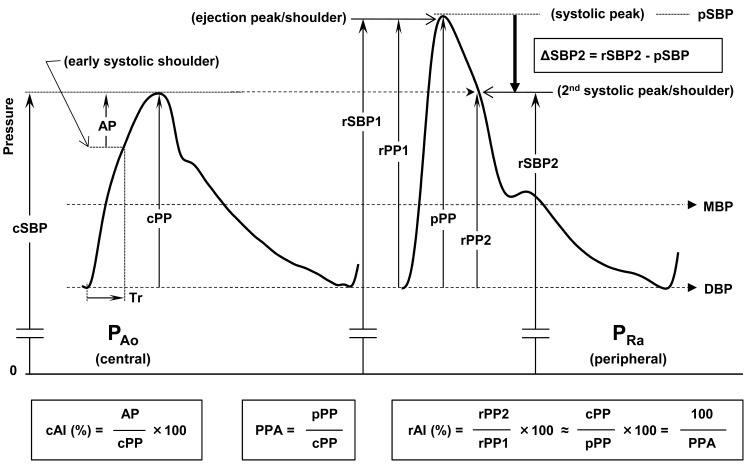
**Central blood pressure (CBP)-related indexes obtained from radial pressure pulse waveform.** Major CBP-related indexes are shown in rectangles with their definitions. AI = augmentation index; AP = augmented pressure; DBP = diastolic blood pressure; MAP = mean arterial pressure; PP = pulse pressure; PPA = PP amplification; rPP2 = pressure amplitude at the second systolic peak or shoulder of radial pressure wave; SBP = systolic blood pressure; rSBP2 = radial late or second systolic pressure; Tr = reflection wave arrival time. Lowercase initials indicate measurement sites: c- = central aortic; ca- = carotid; p- = peripheral; r- = radial.

**Fig. (5):**
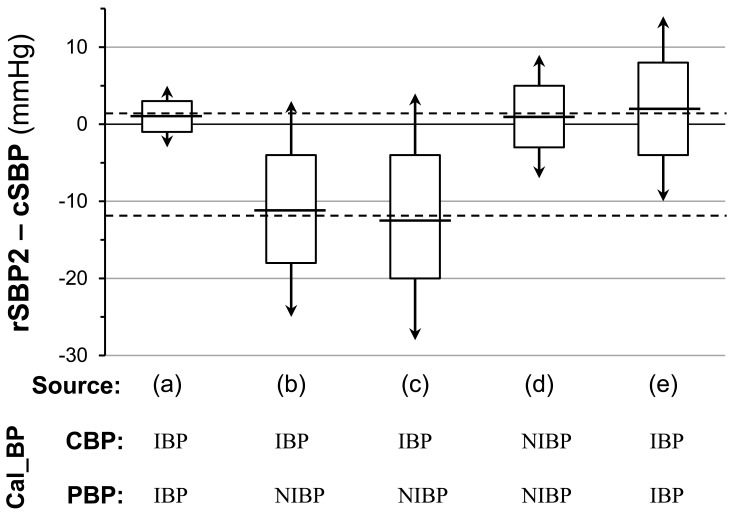
**Reported comparisons between central aortic systolic pressure (cSBP) and radial second systolic pressure (rSBP2).** The mean differences between rSBP2 and cSBP reported in three papers [34-36] shown in Table **2** are plotted on the same plane of coordinates with ranges of ±SD of the differences (rectangular height) and limits of agreement (double-arrowed vertical line). The distance between the two horizontal dashed lines may correspond to the pressure difference attributable to the difference between central (CBP) and peripheral (CBP) BPs in calibration pressures (Cal_BP); i.e., noninvasive (NIBP) vs. invasive (IBP).

This method is based on observations that rSBP2 (the pressure at the second systolic peak or shoulder; Fig. **4**) is nearly identical [34] to or closely correlated [35] with cSBP. Pauca *et al*. [34] measured both central and peripheral BPs directly with fluid-filled manometers and paper chart recordings. On the other hand, Takazawa *et al*. [35] compared noninvasive rSBP2 using radial artery tonometry calibrated to cuff brachial BP with invasively measured cSBP by the use of a micromanometer-tipped guidewire. Later, using data from a large population (N >10,000) from the Anglo-Cardiff Collaborative Trial, Hickson *et al*. showed that noninvasive rSBP2 and cSBP derived from GTF-based aortic pressure waveform estimation using the same radial artery tonometry waveform calibrated to cuff brachial BP, were almost identical except for a trend toward underestimation in rSBP2 for the lower SBP range [36]. Furthermore, Hickson *et al*. also indicated, in their sub-study, the practical equivalence of invasive micromanometric cSBP and rSBP2 calibrated to the same invasive MAP/DBP. These reported findings can be summarized by saying that, if the calibration of central and peripheral waveforms is common, cSBP and rSBP2 are almost equivalent (Table **2**). By taking these findings into account, we find surprisingly that calibration differences may be the main cause of the consistent bias of rSBP2, which was as large as 12 mmHg (peripheral<central) against cSBP, reported by Takazawa *et al*. [35] (Fig. **5**). 

Based on the linear relationship between noninvasive rSBP2 and invasive (actual) cSBP such as Takazawa *et al*. [35] reported, the HEM-9000AI^®^ device estimates and displays cSBP, which is comparable to that of invasive measurement. This implies simultaneous compensation for calibration differences between invasive and noninvasive measurements and for the systematic bias of SBP2 by a single regression model. This compensation can reduce the consistent bias but has no effect on the variance of errors. The large correction sometimes induces the *illusion* that cSBP is higher than peripheral SBP, which is inconsistent with the physiological PPA phenomenon. The inability to determine cPP in the absence of a cDBP estimate that is comparable to the measurement obtained invasively sometimes confuses users.

Additionally, a comparison between cSBP and the peripheral SBP2 of a digital artery pressure waveform measured with a noninvasive volume clamp method, which was also reported to be comparable to the tonometric radial pressure waveform [37], has been reported [38]. In that study, the pressure calibration (invasive/noninvasive) for both central and peripheral waveforms was unified, and a similar relationship (i.e., equivalence) between cSBP and peripheral (finger) SBP2 was shown.

### Comparison Between GTF- and SBP2-based Methods

In contrast to the GTF-based method, which has a relatively clear theoretical basis in relation to both physics and engineering, the SBP2-based method is justified only by “empirical” observations of the equivalence of rSBP2 and cSBP as described above, without a clear explanation or theory about its underlying mechanism. As an interpretation of the second peak of a radial artery pressure waveform, the following somewhat conceptual explanation has been generally accepted [13].

“Aortic reflection waves returning from systemic reflection sites (predominantly from the lower body) to the central aorta usually generate an augmentation peak as the secondary peak of the aortic pressure wave. The augmentation peak is predominantly composed of lower-frequency components, which are not largely influenced by amplification or attenuation during its travel down along the upper limb artery to make the second peak or shoulder of the pressure wave at the radial site.”

Hence, the second systolic peak is also called the “reflection peak”. However, there have never been demonstrative data on which the above explanation can rely. This might relate to the practical difficulty of acquiring a simultaneous flow waveform with a pressure wave. Precise flow wave measurement is essential for analyzing refection waves separately [39]. 

**Fig. (6):**
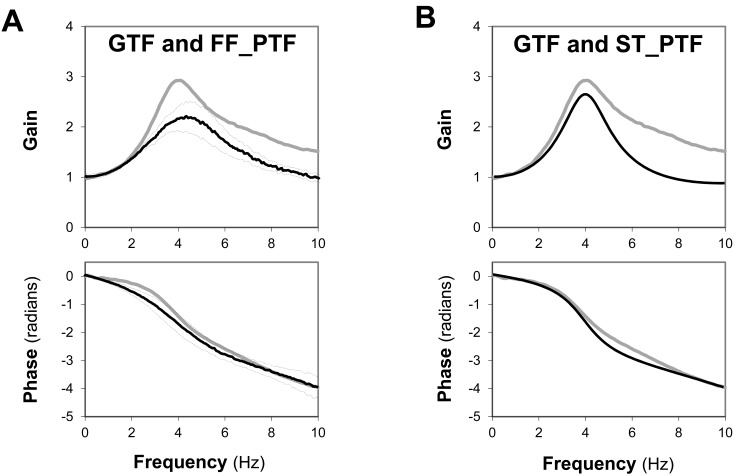
**Similarities of GTF to a fluid-filled pressure system property (FF-PTF; panel A) and to a single elastic tube model property (ST-PTF; panel B).** The thick gray lines represent the gains and phases of the SphygmoCor^®^ GTF as a function of frequency in each panel. In panel A, a pooled PTF (gains and phases) obtained from 8 fluid-filled catheters connected to a clinically used manometer system with rescaling of the frequency axis to adjust peak gain frequencies (solid black line) is superimposed on GTF. The broken lines indicate the range of ±SD. Likewise, in panel B, a calculated PTF (gains and phases) derived from a single elastic tube model is shown in black. The model parameters are grossly determined so that the peak gain and its frequency become close to the characteristics of GTF. In addition, the linear phase delay (i.e., time delay) of each superimposed PTF is also adjusted to that of GTF.

Karamanoglu *et al*., who first proposed GTF [28], reported a detailed simulation study based on a realistic multi-branched model representing the human upper limb arterial system [40] for theoretical validation of the generalizability of an upper limb pressure transfer function. While in some ways their model seems quite complex, the elemental arterial segment was represented simply by a single elastic tube with reflection. Stergiopulos *et al*. employed a similar model focusing on peripheral arterial pulse wave transduction including the upper limb arteries, and their results suggested that the arterial path between central aortic and brachial sites could be simulated by a single loss-less elastic tube model [41]. The term “loss-less” means pressure wave propagation without attenuation, and also it suggests a possibility of an under-damped system with resonant oscillation comparable to a fluid-filled pressure transducer system. In fact, GTF, which is regarded as a representative property of pressure pulse transduction along the upper limb arteries, is surprisingly superimposable on a modified PTF measured from a fluid-filled catheter manometer system simply by rescaling of the frequency axis (Fig. **6A**) as well as on a calculated PTF based on a single elastic tube model (Fig. **6B**), although the peak gain values are somewhat different (unpublished data). 

These findings suggest that what one can see as a radial pressure waveform is a measured central pressure waveform largely distorted through a considerably imperfect fluid-filled pressure line—i.e., the upper limb arteries—and also that the GTF-based estimation method may act as a compensation filter for this distortion. In addition, it is unlikely that only the central augmentation peak travels along the fluid-filled pressure line without distortion. Hence, the mechanism responsible for the fact that rSBP2 is nearly equivalent to cSBP remains to be identified by real data in future studies.

The SBP2 method has been criticized for working well only when the second shoulder of the radial pressure wave is detectable automatically, and for failing to identify the second shoulder 10% of the time [13, 42]. The manufacturer of the device (HEM-9000AI^®^), which employed this method, argues that it has overcome the limitation by an improved detection method (details have not been publicized) that is different from the original method [43]; in the original method, the second shoulder is determined simply based on the third zero cross of the fourth derivative of the radial pressure waveform. Therefore, the timing of rSBP2 demonstrated in a published paper [44] is somewhat different from that determined by the original method [43] and by SphygmoCor^®^ software [42], with only minimal differences in pressure value. The differences among devices in the success rate of rSBP2 determination, shown in Table **2**, may reflect the technical improvement.

## USEFULNESS OF CBP-RELATED INDEXES

The clinical significance of CBP assessment is to evaluate what is impossible to know by cuff brachial pressure measurements. Pressure values such as MAP and DBP, which are common throughout a conduit artery from central to peripheral [2], need not be assessed as CBP. All the differences between central and peripheral pressures exist in the pulsatile components of BP, which are attributable chiefly to reflection wave dynamics. Pulsatile pressure parameters include augmented pressure (AP) due to aortic wave reflection, PP (total pulsation amplitude), and SBP (positive deflection of pulsation with the offset of MAP) as shown in Fig. (**4**). PPA assesses the difference between peripheral and central PP as a ratio, which requires no pressure calibration. The augmentation index (AI) is defined as the fraction of pressure augmentation by aortic wave reflections in PP at the central site (cAI). The AI at the radial site (rAI), however, does not directly reflect cAI because rAI is defined as the ratio of rPP2, which is regarded as a good alternative to cPP, to pPP. This implies that rAI corresponds to the inverse of the PPA [38] when rSBP2 reflects cSBP, i.e., when cAI ≥0% [45], and when the early peak of the radial pressure wave determines pPP, i.e., when rAI ≤100%. Hence, rAI is indirectly related to cAI through its close correlation with PPA [46]. These indexes expressed as ratios do not depend on pressure calibration, whereas they are not useful for evaluating absolute pressure values such as assessing antihypertensive therapy. In a recent study [47], an index named “ΔSBP2” was used to evaluate the central effects of various antihypertensive drugs. The index is simply a central pressure decrease from peripheral SBP determined by subtracting rSBP from rSBP2 that is an estimate of cSBP. This means that MAP is subtracted as the offset and could effectively extract the central effects of antihypertensives.

## ATTEMPTS TO INDIVIDUALIZE CBP ESTIMATION

Even the GTF method, which derives a full waveform of central aortic pressure to estimate CBP values, is insufficient for estimating precise central waveforms or parameters relating to higher-frequency components such as AI, as shown by the validation studies discussed above [14, 31, 32]. To enable precise CBP waveform estimation, some researchers have attempted to individualize transfer-function-based estimation methods [48-50]. None of them, however, succeeded in the practical improvement of estimation accuracy. Karamanoglu, who has proposed the GTF-based CBP estimation method, also comprehensively investigated the digital artery pressure pulse as a peripheral pressure waveform to be used for customized or individualized PTF-based central aortic pressure estimation [51]. However, this measure has not been adopted in any existing dedicated device for CBP estimation, perhaps at least partly because the method for customization is somewhat complex. That is, it requires precise, simultaneous measurement of the carotid pressure waveform, which has aforementioned issues in regard to tonometric recordings, and the digital artery pressure pulse.

It is notable that a novel method, called the “adaptive transfer function”, was proposed [52]. It is based on a new modeling of the arterial system; instead of using a conventional single elastic tube, it uses parallel tubes to model all peripheral arteries branched from the aorta as parallel elastic tubes arising at the same aortic root but not from the branching sites. Then, simultaneous equations about two different transfer functions (TFs) are considered for a single artery of interest; i.e., one is a pressure-input/pressure-output TF and the other is a flow-input/pressure-output TF. One can obtain individual TF parameters by solving the equation. However, it remains to be validated clinically.

## CONCLUSION

No method can be perfect in CBP estimation. Currently, GTF- and SBP2-based methods are the two major methods of estimating CBP noninvasively. They may be even in the accuracy with which they estimate CBP parameters if central and peripheral pressure calibrations are unified. The radial artery may be the optimal site for arterial applanation. Precise as well as highly reproducible applanation tonometry recordings are important for accurate CBP estimation. It should be considered that CBP estimation methods are issues of software that is totally independent of device hardware, *per se*. Hence, how precise the peripheral pressure wave we acquire is more important than which CBP estimation algorithm we select. From this viewpoint, the superiority of the automated arterial tonometry device equipped with an automatically controlled sensor array such as HEM-9000AI^®^ is manifest. Extensive automation of the measurement/estimation procedure will also facilitate the application of CBP estimation to routine clinical practice. 

## Figures and Tables

**Table 1.** Features of Central Blood Pressure Estimation Devices/Methods

Device	Site of Measurement	Measurement Principle	Sensor Structure (Operation)	Calibration (NIBP Measurement)	CBP Estimation Method	Estimated CBP Parameters (CBP Related Indexes)*
**Any tonometric devices**	common carotid artery	applanation tonometry	single (manual)	BrBP: MBP/DBP (manual input of separately measured BrBP)	simple substitution	caSBP, caPP (caAI, PPA)
**SphygmoCor^®^**	radial artery	applanation tonometry	single (manual)	BrBP: SBP/DBP (manual input of separately measured BrBP)	GTF	P_Ao_, cSBP, cDBP, cPP (cAI, AP, PPA)
**HEM-9000AI^®^**	radial artery	applanation tonometry	arrayed (automated)	BrBP: SBP/DBP (automatically measured BrBP with an inbuilt oscillometric sphygmomanometer)	SBP2	cSBP, rSBP, rPP2 (rAI ≈ PPA^-1^%)
**BPro^®^ + A-PULSE CASP^®^**	radial artery	modified applanation tonometry	Single (fixed)	BrBP: SBP/DBP (acquired from a dedicated oscillometric sphygmomanometer temporarily connected before use)	NPMA	cSBP, (rAI ≈ PPA^-1^%)
Dedicated oscillometric devices**	brachial artery	modified oscillometry	brachial cuff (fixed)	BrBP: SBP/DBP (a same brachial cuff is used for BrBP as well as oscillometric pulse wave measurements)	dedicated software**	cSBP, cDBP, cPP (brAI, cAI, AP, PPA)

AI = augmentation index; BrBP = brachial cuff blood pressure; DBP = diastolic blood pressure; MBP = mean blood pressure; NIBP = noninvasive blood pressure; NPMA = N-point moving average; P_Ao_ = aortic pressure waveform; PP = pulse pressure; PPA = PP amplification; rPP2 = pressure amplitude at the second systolic peak or shoulder of radial pressure wave; SBP = systolic blood pressure; SBP2 = late or second systolic pressure of peripheral pressure wave; br- = brachial; c- = central aortic; ca- = carotid; r- = radial.

* Refer to Fig. (**4**) for the relationship between each parameter and blood pressure waveforms.

** They include, e.g. Arteriograph^®^, BPPlus^®^+VasomonR^®^, BPLab^®^+Vasotens^®^, and Mobil-O-Graph^®^ etc.

**Table 2.** Reported Comparisons Between Central Systolic Blood Pressure (cSBP) and Radial Second Systolic Blood Pressure (rSBP2)

Source		Pauca AL *et al.*, 2004 [34]	Takazawa K *et al.*, 2007 [35]		Hickson SS *et al.*, 2009 [36]	
Study*		CABG^ (a)^	baseline^ (b)^	drug intervention ^ (c)^	noninvasive^ (d)^	invasive ^(e)^
**Subjects**		treated IHD/HT pts	cardiac cath. IHD pts	cardiac cath. IHD pts	selected from ACCT cohort	cardiac cath. pts
**Measurement condition**		anesthesia	incl. treated CVD**	nicorandil iv	incl. treated CVD**	incl. treated CVD**
**Total n**		50	18	18	10269	38
**Age (years)**		41-87 (70% of pts >60)	61±10	61±10	60±20	60±9
**rSBP2 determination**	Successful n	21	16	16	10082	34
	Success rate	47%	100%	100%	98%	90%
	Determination method/device	inspection	HEM-9000AI^®^	HEM-9000AI^®^	SphygmoCor^®^	SphygmoCor^®^
**Other exclusion (n)**		5	2	2		
**Measurement method (device)**	cSBP	invasive (FF-cath)	MM-GW (PressureWire^®^)	MM-GW (PressureWire^®^)	GTF-based estimation (SphygmoCor^®^)	invasive (MM-cath)
	rSBP2	invasive (FF-cath)	tonometry (HEM -9000AI)	tonometry (HEM -9001AI)	tonometry (SphygmoCor^®^)	tonometry (SphygmoCor^®^)
**Calibration**	cSBP	invasive (FF)	invasive (MM)	invasive (MM)	noninvasive (BrBP)	invasive (MM)
	rSBP2	invasive (FF)	noninvasive (BrBP)	noninvasive (BrBP)	noninvasive (BrBP)	invasive (MM)
**Correlation**	r	NA	0.95	0.93	0.99	0.92
	p	NA	<0.001	<0.001	<0.001	<0.001
**B-A plot difference**	Mean (mmHg)	1	-11	-12	1	2
	SD (mmHg)	2	7	8	4	6

ACCT = Anglo-Cardiff Collaborative Trial; B-A plot = Bland-Altman plot analysis; BrBP = brachial cuff blood pressure; cath. = catheterization; CVD = cardiovascular disease; FF = fluid-filled; FF-cath = FF catheter-manometer; IHD = ischemic heart disease; iv = intravenous administration; MM = micromanometer; MM-Cath = MM-tipped catheter; MM-GW = MM-tipped guidewire; NA = not available; pts = patients.

* Superscripts (a) ~ (e) correspond to those in Fig. (5).

** Including chronically treated patients with cardiovascular disease.
